# Medicine

**Published:** 1900-12

**Authors:** J. Michell Clarke


### Progress of the Medical Sciences.

### MEDICINE.

**Myasthenia Gravis.** Drs. Harry Campbell and Edwin Bramwell in their paper on this disease[1] point out that although about sixty cases have been described, it has attracted but little attention in this country. The chief features are weakness, sometimes amounting to complete paralysis of the voluntary muscles; especially characteristic is the loss of power after voluntary action, to be regained after rest, except in severe cases, and the so-called "myasthenic reaction" of the affected muscles, by which is meant their rapid exhaustion uuder faradic stimulation. Muscles which are in constant action are most apt to be affected, such as the cervical and the eye-muscles. On account of the frequency of implication of the muscles innervated from the bulb, the disease has also been called "asthenic bulbar paralysis." Sensation is unaltered. Both sexes are about equally affected, but the disease comes on at an earlier age in women (average 24 years) than in men (average 35 years). Not unfrequently it has followed upon some acute affection, such as influenza, scarlet fever, typhoid fever, otitis media; occupation, syphilis, alcohol, neuropathic inheritance seem to play no special part in its causation. The mode of onset is usually gradual, and the muscles first affected are those innervated by the bulbar nuclei;

[1] *Brain*, 1900, xxiii. 277.

thus ptosis, diplopia, weakness of lips, indistinct speech, difficulty in mastication and in swallowing, and inability to hold the head upright, have each occurred as the first symptom, but in other cases the limbs have been earliest affected. In addition to the bulbar symptoms given above, complete external ophthalmoplegia, irregular nystagmoid movements, readily induced fatigue of the ocular muscles, weakness of those about the mouth, difficulty of swallowing—in several cases death has followed fits of choking,—weakness of the palate and of the tongue, feelings of fatigue, of aching and stiffness of the tongue, are common symptoms. The face is immobile, expressionless with drooping upper eyelids, and the authors compare the facial expression to that of the Landouzy-Déjèrine form of myopathy. Speech is characteristic. "The patient speaks naturally when he begins, but soon speech becomes nasal; words become more and more indistinct; the voice becomes weaker and weaker, and finally he has to stop for want of breath . . . These peculiarities are well demonstrated when the patient reads aloud."

The muscles of the trunk and limbs are also involved; from weakness of the neck-muscles there may be difficulty in supporting the head; from weakness of the back and abdominal muscles the patient may be unable to sit up long, or to raise himself into the sitting posture without using the hands—in some cases there is the greatest difficulty in turning in bed. Dyspnœa on slight exertion is present when the respiratory muscles are affected. Attacks of breathlessness occur from no apparent reason, and are dangerous to life. If the attack comes when he is walking, the patient falls to the ground cyanosed and breathless. Respiration is noisy, saliva accumulates in the mouth and can neither be swallowed nor expectorated. If lying in bed he sits up and saliva flows from his mouth. Strümpell, who first described them, believes that these attacks are due to sinking back of the tongue. In the limbs the muscles nearest the trunk are most affected—in the legs usually the ilio-psoas and quadriceps extensor; in two cases, however, weakness first appeared in the fingers. When the legs are very weak the gait is waddling, but in slight cases it may be natural, the patient merely having to rest for a minute or two from time to time. Sudden giving way of the legs is often present. When the patient writes, the writing is at first good and rapid, but it soon becomes slower and the letters badly formed, until the attempt has to be relinquished.

The muscles react in a peculiar way to the faradic current—they contract briskly at first to a tetanising current, but if the electrodes are kept in contact, very shortly the muscle ceases to respond; if the electrodes are applied again after a few minutes' rest the muscle again contracts, and again the contraction passes off. This is the **myasthenic reaction.** It is not present in all cases, it occurs only occasionally; it is met with in different muscles at different times in the same case, and in the same muscle there is great variability in the ease with which it can be produced.

Muscular atrophy has been met with in a few cases, but it is "altogether exceptional." Fibrillary contraction was present in the tongue in two cases. The sphincters are never affected. The muscular sense, co-ordination, superficial reflexes are normal; sensory symptoms are absent, except for a sense of fatigue or stiffness in the affected muscles. The knee-jerks are generally more active than normal. Great variations occur in the intensity of the symptoms from time to time, and are characteristic of the disease. "The most striking feature is the speedy production of muscular exhaustion." Emotional excitement, exposure to cold, the catamenial periods in women, increase the symptoms.

In the majority of cases no visible lesion has been discovered after death. The pathology of the disease is obscure, but the authors suggest that it is due to the circulation in the blood of a toxin, probably of microbic origin, which acts selectively upon the lower motor neuron, so as to modify its functional activity.

As to prognosis, 23 out of 60 recorded cases died, the average duration of illness in the fatal cases being a year and a half; but some cases improve in a remarkable way, and have been known to remain free from symptoms for months, or even years, and it is probable that complete recovery sometimes occurs. Involvement of the respiratory muscles is a very grave symptom.

No specific treatment is known: an abundant and nutritious diet, and avoidance of cold and fatigue are important points; iron and arsenic are of use as general tonics; strychnine has been tried without benefit.

**Subacute Combined Degeneration of the Spinal Cord.** This paper, by Drs. Risien Russell, F. E. Batten, and James Collier,[1] is based on the study of nine cases. The authors propose the above name provisionally, understanding by it a disease in which tracts of different function are concomitantly affected, and one which possesses a very definite clinical picture, not as a rule difficult of recognition, and a characteristic morbid anatomy. Obviously the term "combined degeneration" applies to other affections, and they prefix "subacute" to differentiate it. The cases first recorded were described by Lichtheim as examples of cord changes associated with anæmia. With regard to published cases the relation of anæmia to subacute combined degeneration of the spinal cord is threefold. These are (1) the cases of subacute combined degeneration of the cord dealt with here, in some of

[1] *Brain*, 1900, xxiii. 39.

which anæmia is a symptom; (2) cases of anæmia in which symptoms of nerve disorder arise and changes are found in the cord after death; and (3) cases of severe anæmia without clinical manifestations of nerve-lesions, but in which, nevertheless, definite morbid changes are found in the spinal cord after death. To take the first group, with which alone the authors deal in their paper, the disease runs its course in three stages, almost always to a fatal issue. They give the following summary:—

1. A stage of slight spastic paraplegia, with slight ataxy and marked subjective sensations in the lower limbs.

2. A stage of severe spastic paraplegia, with marked anæsthesia of legs and trunk.

3. Stage of complete flaccid paraplegia; absent knee-jerks; absolute anæsthesia; rapid wasting and loss of faradic excitability in the muscles of the paraplegic region; increase of superficial reflex excitability; absolute incontinence of both sphincters and œdema of the lower extremities and trunk.

They do not include anæmia as a typical symptom, because it was absent throughout in some of the most typical cases; when anæmia occurred, the changes in the blood, &c., were not those of pernicious anæmia. The disease appears in the 4th and 5th decades of life, and the patients were without exception strong and healthy before the symptoms showed themselves. Syphilis was present in four, alcoholic excess in three, and prolonged suppuration in three other cases. The onset in most was slow and insidious, but in the most acute cases rapid, ushered in by headache, vomiting, pyrexia, and malaise. The clinical picture of **Stage 1** was that of slight ataxic paraplegia. The first symptoms were subjective sensations of numbness, stiffness, and sometimes tingling in the lower extremities, followed either quickly or after some months by stiffness and clumsiness of the legs, and some dragging of the feet in walking. Objectively there were increase of deep reflexes in the legs, extensor response in the plantar reflex, and slight loss of sense of position in the legs. Similar symptoms and signs appear in the upper extremities, generally at a later date, and first noticed in writing. The cranial nerves and sphincters remain unaffected.

The **second stage** is ushered in abruptly by loss of power to stand or walk, and anæsthesia in the legs and lower part of the trunk. In five cases this change occurred in a single night. The most characteristic sign of this stage is the rapid development of anæsthesia, including loss of the sense of position, to which, and not to loss of motor power, the inability to stand or walk was due. Though motor power steadily declined, it was never completely lost until the final stage. The muscles were rigid, did not waste, and gave normal electrical reactions. Tabetic athetosis was usually present in the hands. At first the anæsthesia of the skin was peripheral in distribution, affecting first the hands and feet, but as it spread upwards it became segmental. The highest point it reached in any case was the upper limit of 6th cervical root area. Severe constant dragging pain below the costal margin on one side was present in every case, a girdle pain was common, and lightning pains frequent. The mental faculties were unaffected; the sphincters escaped, or were only slightly affected. The cranial nerves (3rd and 7th) were involved in some cases. Reflex iridoplegia was never observed. The superficial and deep reflexes were increased, and jaw- and foot-clonus obtained. The lower lumbar and first sacral areas were always affected before the lower sacral and upper lumbar, and the lower cervical segments before the upper thoracic. There were no trophic changes. Irregular elevations of temperature occurred from time to time, irrespective of the presence of anæmia. The length of this stage was less than one-sixth of whole illness, three weeks to six months, average five weeks.

The **third stage** came on rapidly, accompanied by a change in the general condition, shown by pyrexia, lasting several days, malaise, drowsiness, anorexia, and general asthenia, and the "following succession of symptoms in the lower extremities:— Absolute flaccid motor paralysis; absolute anæsthesia; complete incontinence of the sphincters; loss of the deep reflexes; rapid wasting of the muscles, with loss of faradic excitability; and œdema of the legs and trunk." In the lower limbs and trunk wasting was general, but in the upper limbs it appeared later, and affected in order the intrinsic muscles of the hands, ulnar flexors, radial flexors, extensor of wrist and of elbow, supinators, and 5th cervical group. The anæsthesia became absolute and spread upwards, reaching as high as the 1st dorsal segment before death, in two cases to the 6th cervical segment. Pain disappeared with exception of the heavy pain beneath the costal margin. Signs of mental disturbance and deterioration appeared, with, in three cases, convulsions in the form of fine clonic spasms affecting the limbs. Severe bed-sores developed. The condition of the cranial nerves was unaltered. The duration of this stage was about six weeks, three weeks being the shortest period, and death was due either to sudden syncope or respiratory failure. The condition in this part of the illness was one "of mental and physical paralysis, profound anæmia, general emaciation, and sepsis." The chief pathological changes were found in the spinal cord, and consisted of two distinct processes: (1) a focal destructive lesion and (2) a system lesion. In (1), in the affected areas swelling, followed by fatty degeneration and absorption of the medullated nerve-sheaths, took place, the axis-cylinders being at first unaltered, then disappearing, a space being thus formed, simply surrounded by the connective tissue of the cord. In (2), the system degeneration, the long tracts exhibited a degeneration similar to that found after transverse lesions of the cord. The stress of the disease fell upon the mid-dorsal region of the cord, where there was very marked destruction of the white matter all round the periphery, involving both the endogenous and exogenous fibres: the posterior columns may here be almost completely sclerosed. On tracing the process up the cord the degenerated areas tended to be limited to the posterior columns and crossed pyramidal tracts, and extended to the upper portion of the medulla. Tracing it downwards, the diffuse degeneration was usually limited in the lumbar region to the crossed pyramidal tracts, with some patches in the posterior columns. In the sacral region degeneration was almost entirely limited to the pyramidal tracts. The authors strongly incline to the view that a toxic agent is responsible for the changes in the spinal cord, and for the anæmia which sometimes accompanies them, this toxin producing a parenchymatous degeneration by its action on the nerve elements of the cord. It should have been said that in the first stage of the disease severe intercostal pain was followed by herpes in two cases, and by hemorrhage corresponding to the distribution of a nerve-root in a third. The paper is beautifully illustrated.

J. Michell Clarke.

